# Noise-induced scaling in skull suture interdigitation

**DOI:** 10.1371/journal.pone.0235802

**Published:** 2020-12-17

**Authors:** Yuto Naroda, Yoshie Endo, Kenji Yoshimura, Hiroshi Ishii, Shin-Ichiro Ei, Takashi Miura

**Affiliations:** 1 School of Medicine, Kyushu University, Fukuoka, Japan; 2 Graduate school of Medical Sciences, Kyushu University, Fukuoka, Japan; 3 Department of Neurology, Osaka City General Hospital, Osaka, Japan; 4 Faculty of Science, Hokkaido University, Sapporo, Japan; Technische Universität Dresden, GERMANY

## Abstract

Sutures, the thin, soft tissue between skull bones, serve as the major craniofacial growth centers during postnatal development. In a newborn skull, the sutures are straight; however, as the skull develops, the sutures wind dynamically to form an interdigitation pattern. Moreover, the final winding pattern had been shown to have fractal characteristics. Although various molecules involved in suture development have been identified, the mechanism underlying the pattern formation remains unknown. In a previous study, we reproduced the formation of the interdigitation pattern in a mathematical model combining an interface equation and a convolution kernel. However, the generated pattern had a specific characteristic length, and the model was unable to produce a fractal structure with the model. In the present study, we focused on the anterior part of the sagittal suture and formulated a new mathematical model with time–space-dependent noise that was able to generate the fractal structure. We reduced our previous model to represent the linear dynamics of the centerline of the suture tissue and included a time–space-dependent noise term. We showed theoretically that the final pattern from the model follows a scaling law due to the scaling of the dispersion relation in the full model, which we confirmed numerically. Furthermore, we observed experimentally that stochastic fluctuation of the osteogenic signal exists in the developing skull, and found that actual suture patterns followed a scaling law similar to that of the theoretical prediction.

## Introduction

Sutures are the thin, soft tissues between skull bones. They perform multiple functions, and they have been extensively studied as a model system of skeletal development [1]. During development, the suture tissue acts as the growth center of the skull; the premature disappearance of the suture tissue causes the skull deformation known as craniosynostosis [2]. At birth, the suture tissue is thick and straight; however, as the skull develops, the sutures gradually becomes thinner and begins to form winding and interdigitated patterns [3]. After adolescence, the skull bones gradually fuse and the suture tissues disappear. Consequently, suture tissue can be used to estimate the age of a person in forensic science techniques [4]. The suture tissue is known to mechanically connect the skull bones, and the interdigitation is assumed to reinforce the mechanical strength of the connection [5].

The interdigitation of sutures results in a fractal structure, which was initially reported in the mid-1980s [6, 7]. Until recently, researchers generally measured the fractal dimension by the box-counting method [8–13]. These measurements have mainly been used for classification or diagnostic purposes; however, studies are yet to reveal the mechanism of fractal pattern formation.

Although various models have been proposed for the formation of fractal structures, e.g., the Eden collision model [14], the Koch curve [15] and diffusion-limited aggregation [16], their application to suture pattern formation has been unsuccessful. For example, (i) Oota et al. [17, 18] applied the Eden model to the formation of the curvature of skull sutures, (ii) Zollikofer and Weissmann applied the diffusion-limited aggregation method to the formation of skull suture interdigitation [16], and (iii) we suggested that the formation of skull suture curvature based on the time-dependent diffusion term was equivalent to the Koch curvature generation [3]. However, these models produced results inconsistent with the experimental observations. For example, in our previous study [3], we introduced time-dependent diffusion terms to reproduce the fractal structure [3]; however, in a subsequent study that used computed tomography, we were unable to detect the addition of small structures to the large structures during development, which is expected in a model with time-dependent diffusion terms [19].

**Fig 1:**
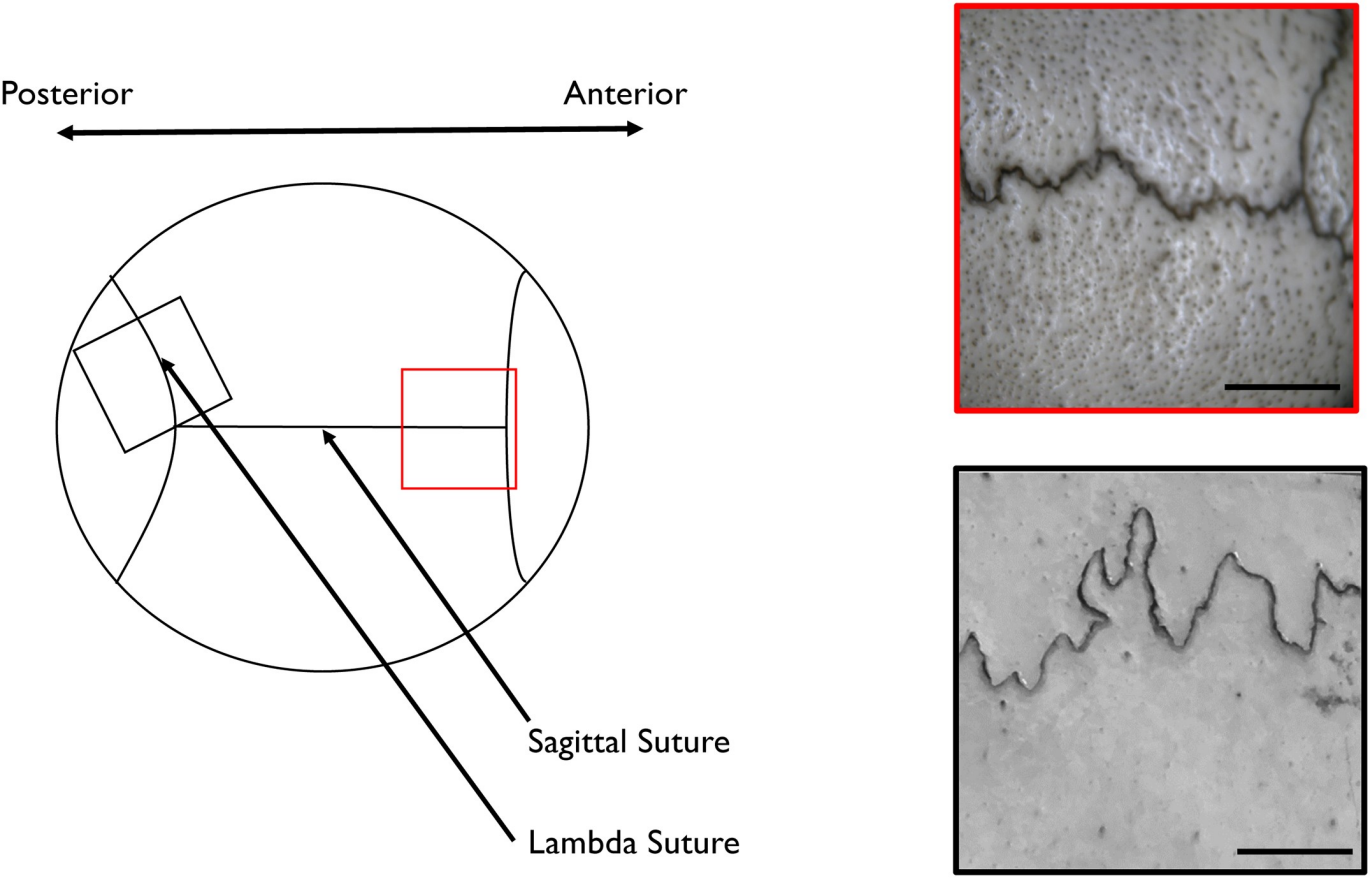
The anterior part of the sagittal suture in an adult skull shows stochastic small amplitude interdigitation. The suture in the anterior skull near the bregma (red box) has a rough surface with less prominent curvature and a more stochastic appearance than that of the lambda suture (black box). Scale bars (bottom right of boxes) = 0.5 cm.

**Fig 2:**
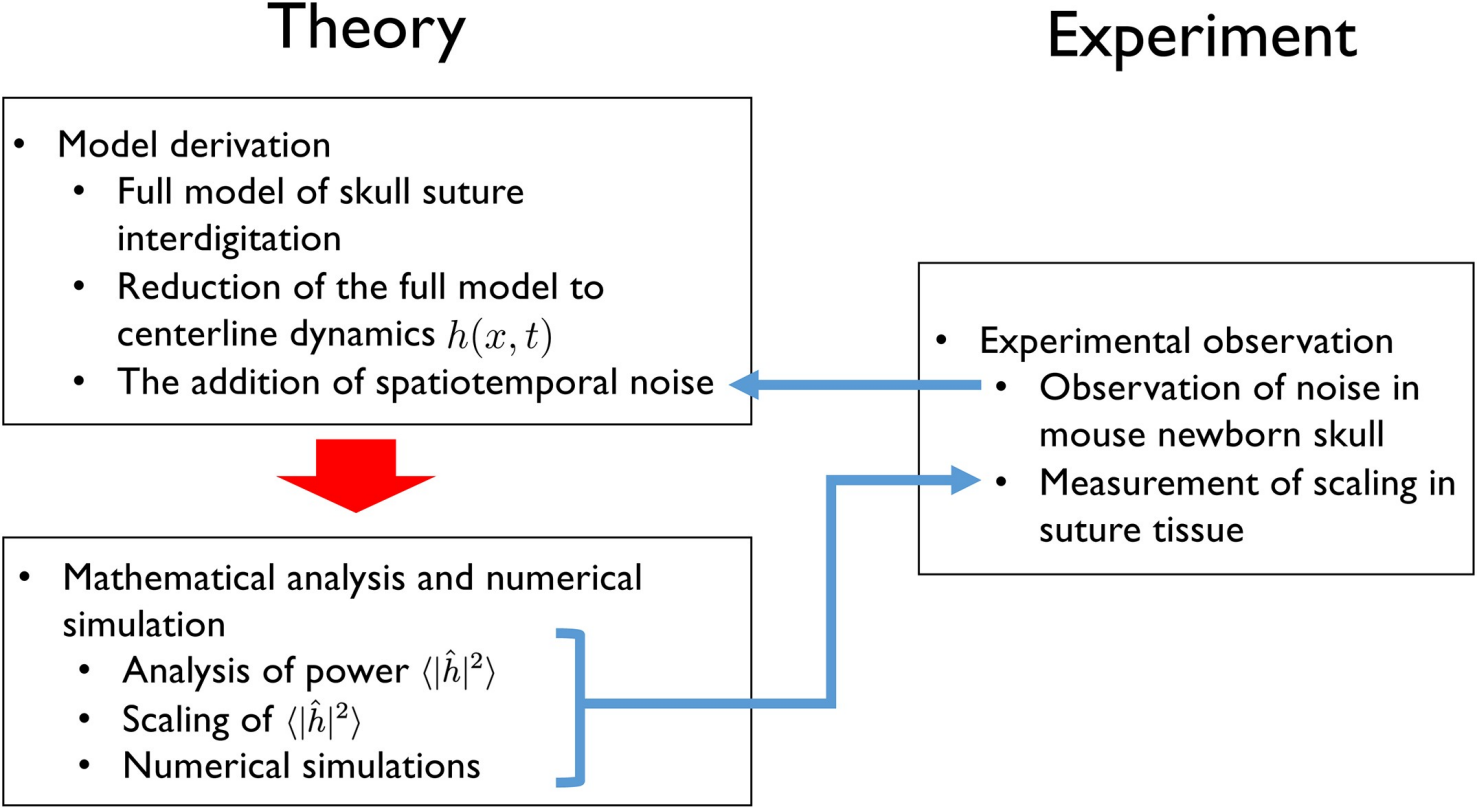
Schematic diagram of the present study. First, we reduced our previous model [19] into graph form *h*(*x*, *t*). Next, we added a noise term to the model based on our experimental observation of noise (Fig 6). Then we mathematically derived the scaling law from this equation and confirmed it using numerical simulations. Finally, we showed experimentally that both noise and scaling do exist in skull sutures (Fig 7).

In the present study, therefore, we propose a new mechanism for suture fractal pattern formation that incorporates a noise term. We focus on the anterior part of the sagittal suture, which exhibits less prominent interdigitation and more random characteristics than other sutures (Fig 1). To understand this pattern formation, we started from our previous model [19] and chose parameter sets which do not show interface instability. Next, we reduced the model to the dynamics of the centerline *h*(*x*, *t*). Subsequently, utilizing previous studies of dynamic scaling [20, 21], we introduced time–space-dependent noise to the model and showed that this system could exhibit scaling. Finally, we demonstrated experimentally that the noise of the osteogenic signal existed in the developing skull, and the scaling of the anterior part of the sagittal suture was consistent with the model predictions. The overall structure of this study is schematically presented in Fig 2.

## Materials and methods

### Observation of noise in newborn mouse skull

To observe spatiotemporal noise in a developing mouse skull, we used a transgenic mouse that expressed a FRET sensor for the phosphorylated ERK signal [22] as well as an organ culture system. We sacrificed newborn mice by decapitation and dissected their skulls using forceps and a scalpel. The isolated skulls were then placed onto Millicell culture inserts (Millipore Inc.) with a Dulbecco’s modified Eagle medium/F-12 culture medium containing 10% fetal bovine serum and antibiotics. We observed the cyan and yellow fluorescent protein ratio (CFP/YFP ratio) using a Nikon A1R confocal microscope, and we quantified the spatiotemporal fluctuation using Fiji software [23]. We have undertaken three independent experiments (three samples from three different litters), and all of them showed similar results. Raw data are accessible via figshare.com (10.6084/m9.figshare.13211339). This experiment was undertaken with the permission of the Kyushu University animal experiment committee (A29-036-1).

### Measurements of human sagittal sutures

Anterior sagittal skull sutures in human bone specimens from Kyushu University School of Medicine and School of Dentistry were digitized using a Stemi 2000-CS stereomicroscope (0.65x magnification; Carl Zeiss) with a digital camera (COOLPIX P6000; Nikon) and an adapter (NY-P6000 Super; Microscope Network Co., Ltd.). Detailed information about these skulls, including their origins, was not available.

We took photographs of the sagittal suture at a distance of 2 cm from the glabella. We then selected 13 sutures with small amplitude interdigitation and without overhang, and we printed images of these sutures onto A4-sized sheets of paper with reduced contrast. For each image, we traced the sagittal suture with a red marker pen and used a document scanner (ScanSnap iX500; Fujitsu) to scan the image into a computer. Using Fiji software [23], we first separated the red channel of the scan to obtain the suture line, which was then skeletonized in order to detect its x and y coordinates. Finally, the coordinate data were mathematically analyzed using *Mathematica* (Wolfram Research). Raw data are accessible via figshare.com (10.6084/m9.figshare.13211339).

### Ethics

This work was approved by Kyushu University Institutional Review Board for Clinical Research (2019-350). Human skull samples used in this study (*n* = 50) were those used for the education of osteology at the Faculty of Medicine, Kyushu University. Since the samples were collected several decades ago and already anonymized, we could not obtain informed consent in this case. Therefore, we notified the general public of study via our laboratory homepage (http://www.lab.med.kyushu-u.ac.jp/anat1/) according to the rules of the local ethics committee.

## Results

### Model derivation

#### Full model of skull suture interdigitation

**Fig 3:**
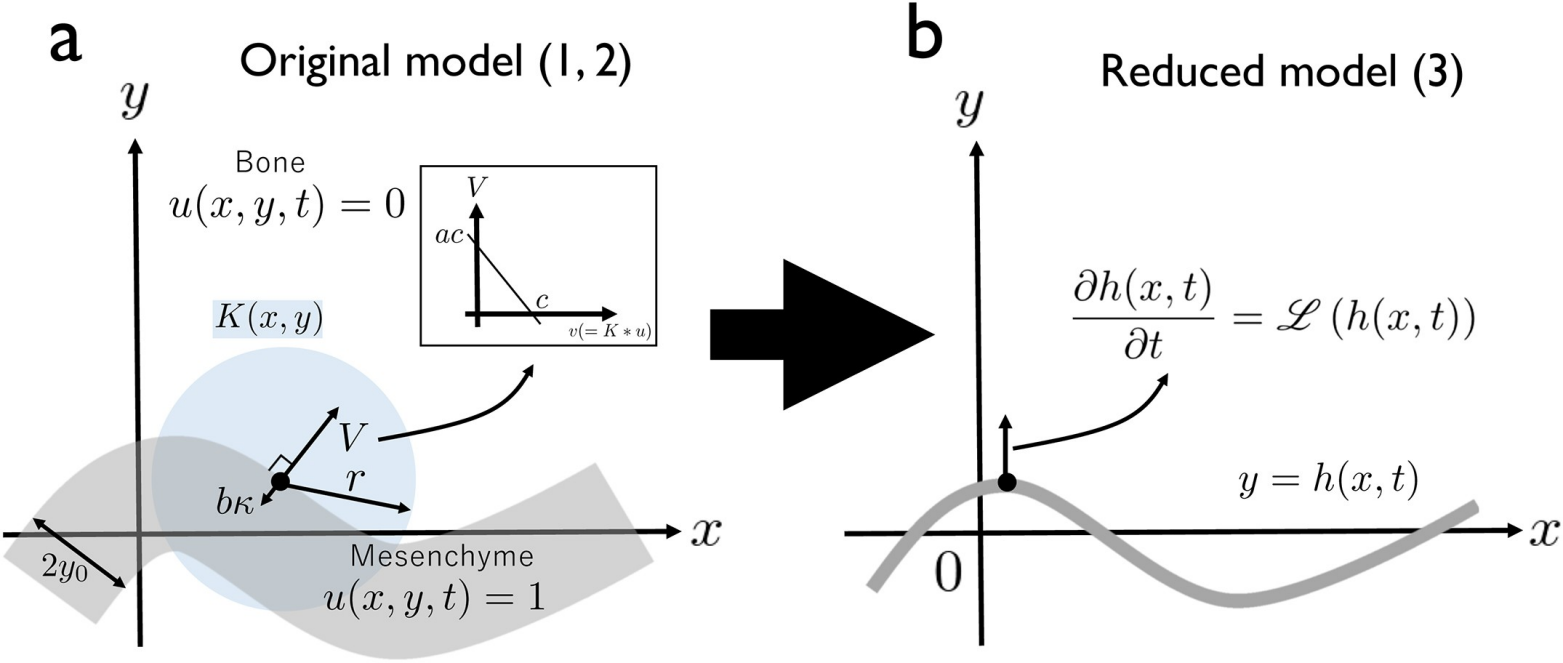
Original model and its reduction. (a) The original model ([19], Eqs (1) and (2)). The model considered a band-like solution with width 2*y*_0_. Growth speed of the interface (*V*) is determined by the effect of substrate molecule (*v*(= *K* * *u*), inlet) and surface tension *bκ*. (b) The present model (Eq (3)). This model focuses on the dynamics of the centerline *h*(*x*, *t*) of the band-like solution, considering only the onset of pattern formation without overhangs.

We previously proposed the following simple model of skull suture interdigitation (Fig 3, [19]):

V=a(c−v)−bκ (1)

v=K*u. (2)

Intuitively, Eq (1) means that the bone differentiation or resorption occurs by the influence of the signaling molecule *v*, and Eq (2) means that the signaling molecule *v*, which is produced by the mesenchyme tissue, has a specific spatial range of action *K*. *V* is the interface speed perpendicular to the bone–mesenchyme interface, *a* is the efficiency of the substrate factor (the osteogenesis-promoting diffusible signaling molecules expressed at the mesenchyme) over bone differentiation, *c* is the threshold value for bone generation/resorption. *b* is the surface tension, and *κ* is the local curvature. In addition, *v*(*x*, *y*, *t*) represents the effect of the substrate factor, determined by the convolution of the kernel *K*(*x*, *y*) and bone shape *u*(*x*, *y*, *t*) (where *u*(*x*, *y*, *t*) = 0 represent bone and *u*(*x*, *y*, *t*) = 1 represent mesenchyme). We used step function with radius *r* for *K*(*x*, *y*) (*K*(*x*, *y*) = 1/(*πr*^2^) when $\sqrt{x^{2}+y^{2}}\leq r$ and *K*(*x*, *y*) = 0 otherwise). This system has a band-like solution, and depending on the parameter set, the solution has interface instability. Its linear dynamics are well understood (see [19] and subsection A. in S1 Text).

Intuitive explanation of suture width maintenance and interdigitation formation in the previous work is as follows [3]: mesenchyme region produce substrate factors *v* which promote osteogenesis, and the concentration of *v* at the bone-mesenchyme interface is dependent on the width of the mesenchyme. When the mesenchyme width is too wide, the osteogenic front proceeds to the point where the concentration is optimal (*v* = *c*). When the mesenchyme width is too narrow, the osteogenic front retracts. As a result, the mesenchyme tissue width is kept constant. In addition, a slightly protruded region should be exposed to a higher concentration of substrate factor *v* since it is surrounded by mesenchyme tissue that produces substrate factors. As a result, when this effect exceeds the effect of surface tension, slight protrusion or convex of bone is amplified and resulting in interdigitation of suture tissue. Mathematically, with a certain parameter set, the real part of the eigenvalue λ(*k*) takes a maximum positive value at *k*_max_, indicating the emergence of a structure with a specific wavenumber (S2 Fig in S1 Text). However, with this model, the fractal structure should not appear.

From the next section, we focused on sections of sutures with less pronounced curvature (red panel in Fig 1). Therefore we used the parameter sets that satisfy λ(*k*) < 0 for all *k* (lower left part of S2 Fig in S1 Text).

#### Reduction of the full model to centerline dynamics *h*(*x*, *t*)

Next, we further simplified the model to represent only the linear dynamics of the full system as follows (Fig 3):

$$\begin{array}{c} \frac{\partial h(x,t)}{\partial t}=ℒ(h(x,t)) \end{array}$$ (3)

where *h*(*x*, *t*) is the *y*-coordinate of the suture at *x*-coordinate *x* and time *t*. Only the small amplitude patterns without overhangs were considered. Here, *ℒ* represents a linear operator that reproduces the linear dynamics of the full model given by Eqs (1) and (2). The explicit form of *ℒ* is described in subsection B. in S1 Text. Thus, the Fourier transformation of *ℒ* should be λ(*k*) given by Eq (14) (subsection A. in S1 Text), and the frequency domain for the system (Eq (3)) is as follows:

$$\begin{array}{c} \frac{\partial \hat{h}\left(k,t\right)}{\partial t}=\lambda \left(k\right)\hat{h}\left(k,t\right). \end{array}$$ (4)

#### The addition of spatiotemporal noise

In this section, we incorporated the noise term *H* in the full system (1, 2) as follows:

V=f(v)−bκ (5)

v=K*u+H(x,y,t). (6)

Here, *H* was defined as the spatiotemporal white noise caused by the known stochastic fluctuation of gene expression ([24], Fig 6).

In the full model, the band-like solution moves according to the *gradient* of the noise $\frac{\partial H}{\partial y}$ (subsection D. in S1 Text). The reduced model considers only changes with small amplitudes without overhangs, neglecting movement in the *x*-direction. Therefore, it can be assumed that the noise term at the suture point (*x*, *h*(*x*, *t*)) is white noise (subsection D. and S6 Fig in S1 Text). The final reduced model with spatiotemporal noise is as follows:

$$\begin{array}{c} \frac{\partial h(x,t)}{\partial t}=ℒ\left(h\left(x,t\right)\right)+\eta \left(x,t\right). \end{array}$$ (7)

We define that *η*(*x*, *t*) is white noise with a mean value of 0 and variance *D*.

$$\bar{\eta (x,t)}=0$$ (8)

$$\bar{\eta {\left(x,t\right)}^{2}}=D.$$ (9)

$\bar{\eta }$ represents the spatial average of *η*.

### Mathematical analysis and numerical simulation

#### Analysis of power $\langle |\hat{h}\left(k,t\right){|}^{2}\rangle$

In a fractal structure, the measurement scale and measured quantity have a linear relationship in log–log plots. For the frequency domain, the wavenumber *k* and power $\langle |\hat{h}\left(k,t\right){|}^{2}\rangle$ should show linearity on log–log plots [25]. This implies that

$$\begin{array}{c} \left\langle \right|\hat{h}\left(k,t\right){|}^{2}\rangle \propto k^{\gamma } \end{array}$$ (10)

holds in a fractal structure; an intuitive explanation of this is presented in subsection C. in S1 Text. The goal of this analysis was to show the relationship in Eq (10).

The Fourier transformation of (3) is

$$\begin{array}{c} \frac{\partial \hat{h}\left(k,t\right)}{\partial t}=\lambda \left(k\right)\hat{h}\left(k,t\right)+\hat{\eta }\left(k,t\right). \end{array}$$ (11)

λ(*k*) represents the linear dynamics of the original model. The concrete form of λ is described in (14) in S1 Text. The steady-state of $\langle |\hat{h}\left(k,t\right){|}^{2}\rangle$ can be obtained by taking the limit as *t* → ∞ [20, 21]:

$$\begin{array}{c} \lim_{t\to \infty }\left\langle \right|\hat{h}\left(k,t\right){|}^{2}\rangle =\frac{-D}{l\lambda (k)}. \end{array}$$ (12)

The sample mean of the $\hat{h}$ variance $\langle |\hat{h}{|}^{2}\rangle$ in the steady state (12) is inversely proportional to λ(*k*). Therefore, when λ(*k*) can be approximated by λ(*k*) ∝ *k*^−*γ*^ at a certain spatial scale, the resulting pattern can be fractal.

#### Scaling of $\langle |\hat{h}\left(k,t\right){|}^{2}\rangle$

If we can approximate λ ∝ *k*^−*γ*^, then the resulting pattern has some scaling. In other words, the log–log plot of (*k*, −1/λ(*k*)) should show linearity, and thereby indicate that λ ∝ *k*^−*γ*^.

**Fig 4:**
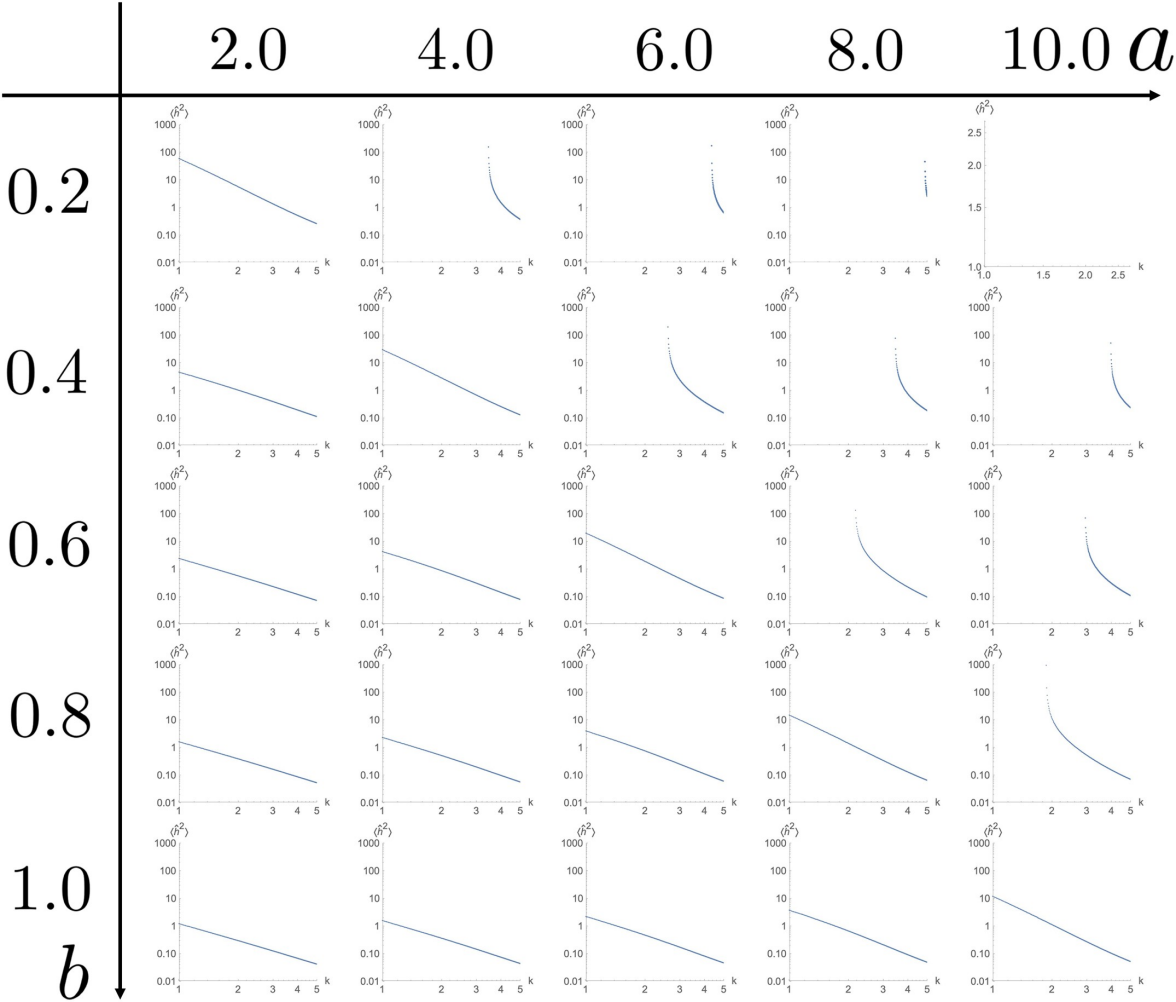
Log–log plot of the (*k*, −1/λ(*k*)) of full model, which should reflect the power spectrum $\langle |\hat{h}\left(k,t\right){|}^{2}\rangle$. The distribution shows linearity in the parameter range without spontaneous pattern formation, indicating λ ∝ *k*^−*γ*^.

We systematically changed the model parameter set (*a*, *b*), and construct log–log plots of (*k*, −1/λ(*k*)). In very large and small spatial scales, scaling was determined by the surface tension term (subsection E. in S1 Text). However, in the parameter regions without interface instability, the plots showed linearity within a certain range of spatial scale (Fig 4, lower left half), indicating that a fractal structure should arise in this parameter region. The gradient varied depending on the parameter set (*a*, *b*).

#### Numerical simulations

**Fig 5:**
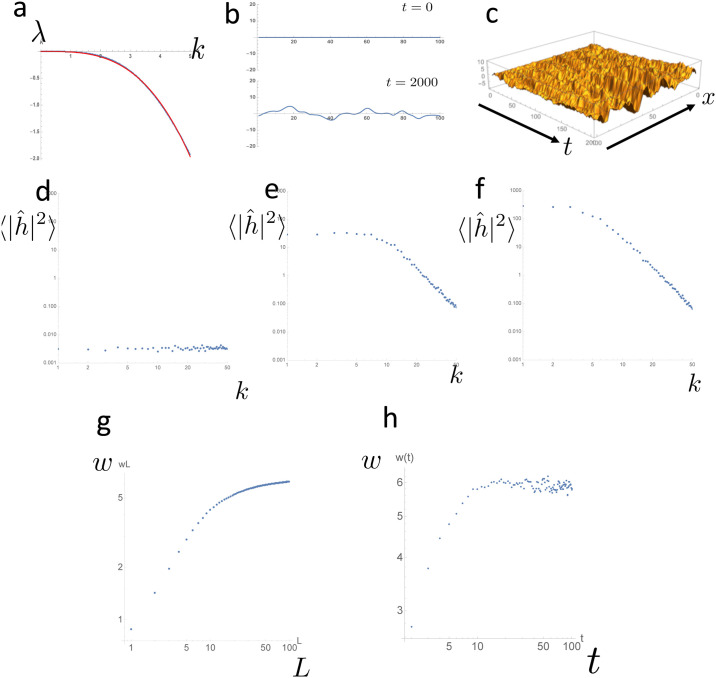
Numerical simulation of the reduced model showed the scaling predicted by the mathematical analysis. (a) Dispersion relation of the full model (blue) and its approximation by λ(*k*) ∝ *k*^*γ*^ (red). The parameter set was *a* = 1, *b* = 0.1, *c* = 0.48, and *r* = 1. Domain size = 20*π* and Δ*x* = 0.2*π*. We defined *H*(*x*, *y*, *t*) as a white noise and obtained $\eta \left(x,y,t\right)=\frac{\partial H}{\partial y}$ at each time step. We used cutoff frequency *ω*_*c*_ = 0.8 to obtain this differentiation. (b) Result of the numerical simulation. The upper panel shows the initial shape (*t* = 0); the lower panel shows the curved shape obtained after sufficient time had passed (*t* = 2000). (c) Log–log plots of the average of ${\hat{h}}^{2}$ obtained by numerical simulations. A region of linear scaling was observed in the high wavenumber region. (d–f) Time course of the log–log plots of the power spectrum obtained by numerical simulations. (d) *t* = 0, (e) *t* = 1000, and (f) *t* = 10000. (g) The relationship between surface roughness *w* and system size *L*. (h) The relationsip between surface roughness *w* and time *t*.

Numerical simulations of our model (Eq (3)) showed that the reduced model could generate patterns with scaling (Fig 5). We initially chose a parameter set that did *not* exhibit interface instability (Fig 5a). The numerical simulation produced a suture pattern (Fig 5b) that resembled the small amplitude interdigitation observed in vivo (Fig 1). Because of the continuous input of noise *η*, the distribution of a single example *h*(*x*, *t*) did not reach a steady-state (Fig 5c). The scaling of the sample mean of the power spectrum 〈|*h*(*x*, *t*)|^2^〉 expected from the mathematical analysis was achieved after a sufficiently long simulation time (Fig 5d–5f). To examine whether the system shows dynamic scaling, we also measured the surface roughness *w*(*L*, *t*), which is defined as

$$\begin{array}{c} w\left(L,t\right)=\sqrt{\frac{1}{L}\sum_{i=1}^{L}{\left(h\left(i,t\right)-\bar{h}\left(i,t\right)\right)}^{2}}. \end{array}$$ (13)

Log–log plot of *w* over *L* and *t* shows power law growth and saturation (Fig 5g and 5h), indicating the existence of growth exponent *β* and roughness exponent *α*.

### Experimental observation

#### Observation of noise in a newborn mouse skull

**Fig 6:**
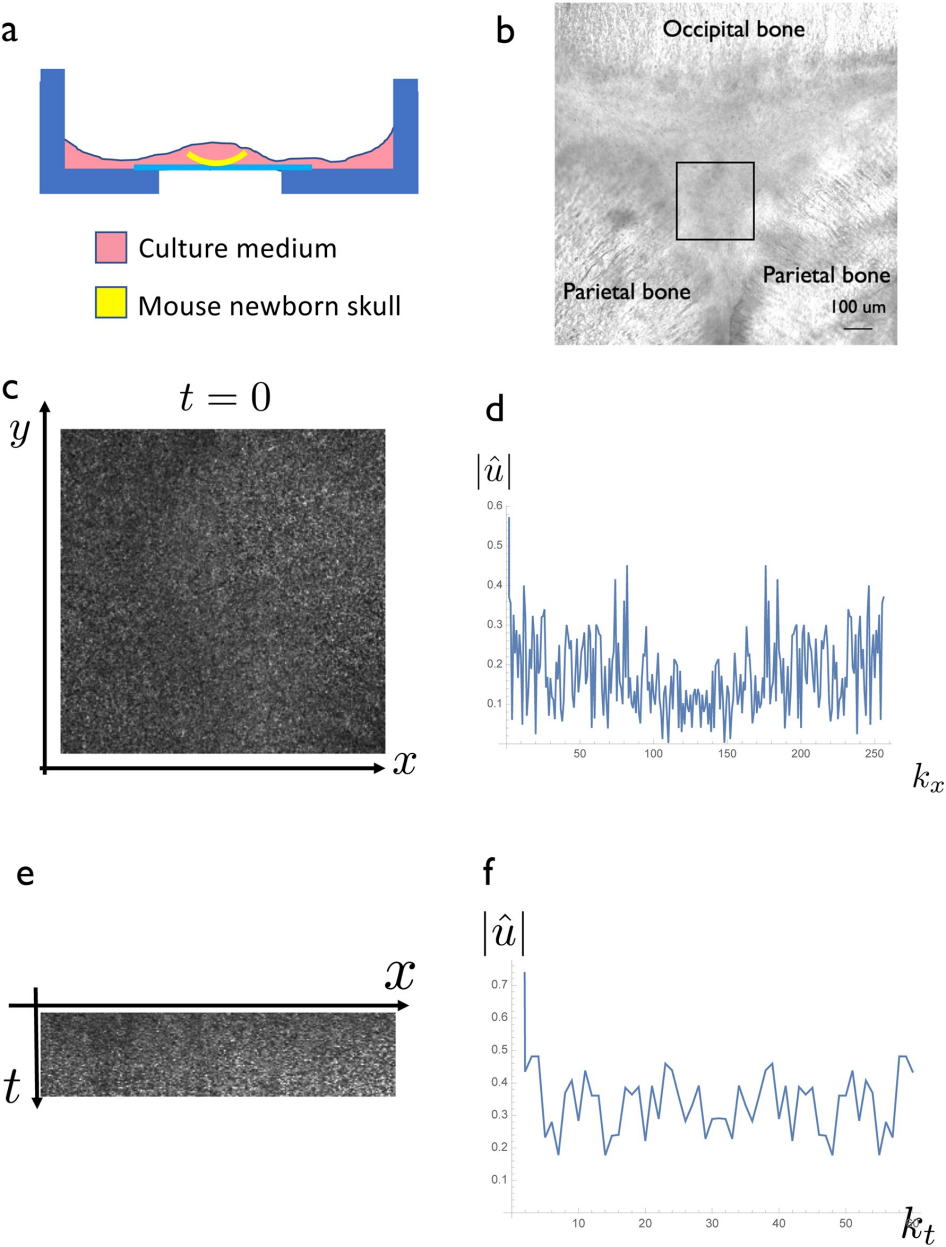
Direct observation of the intrinsic noise of the osteogenic signal in a newborn mouse skull. (a) Experimental system setup. A newborn skull of an ERK-FRET transgenic mice was dissected and then set on the glass bottom dish, which was suitable for organ culture. (b) Brightfield Image of the observation area. We chose posterior fontanelle region for observation. Observation areas in (c-f) is shown by black box. Scale bar = 100 *μm*. (c) Spatial distribution of the FRET signal *u* (CFP/YFP ratio) at specific timepoint. (d) Fourier transformation of *u*(*x*, *t*). There was no specific characteristic in the distribution, indicating the noise was white noise. (e) Time course of FRET signal intensity at a specific line. The signal showed fluctuation. (f) Fourier transformation of (e) at single point. No specific trend was observed, indicating that the spatial noise could be regarded as white noise.

To justify the introduction of noise term *η* in the governing Eq (7), we observed ERK signal input fluctuation using ERK-FRET mice [22]. We use mice model for this experiment since pattern formation dynamics and molecular pathways in mice and humans are similar, especially at the onset of suture pattern formation [3], and it is impossible to directly use a human fetus for an experiment for ethical reason. One of the major signals for osteogenesis is the FGF pathway [3], and the phosphorylation of ERK should represent the signal input of this FGF pathway [22]. We set up an organ culture system of the newborn mouse skull expressing ERK-FRET sensor (Fig 6a) and observed the spatiotemporal signal fluctuation (Fig 6b). We observed the spatial and temporal fluctuation of the ERK input signal (Fig 6a–6d). The power spectrum of spatial and temporal noise seemed flat (Fig 6c–6f), indicating that the observed noise was white noise as assumed in the model (7).

#### Measurement of scaling in human suture tissue

**Fig 7:**
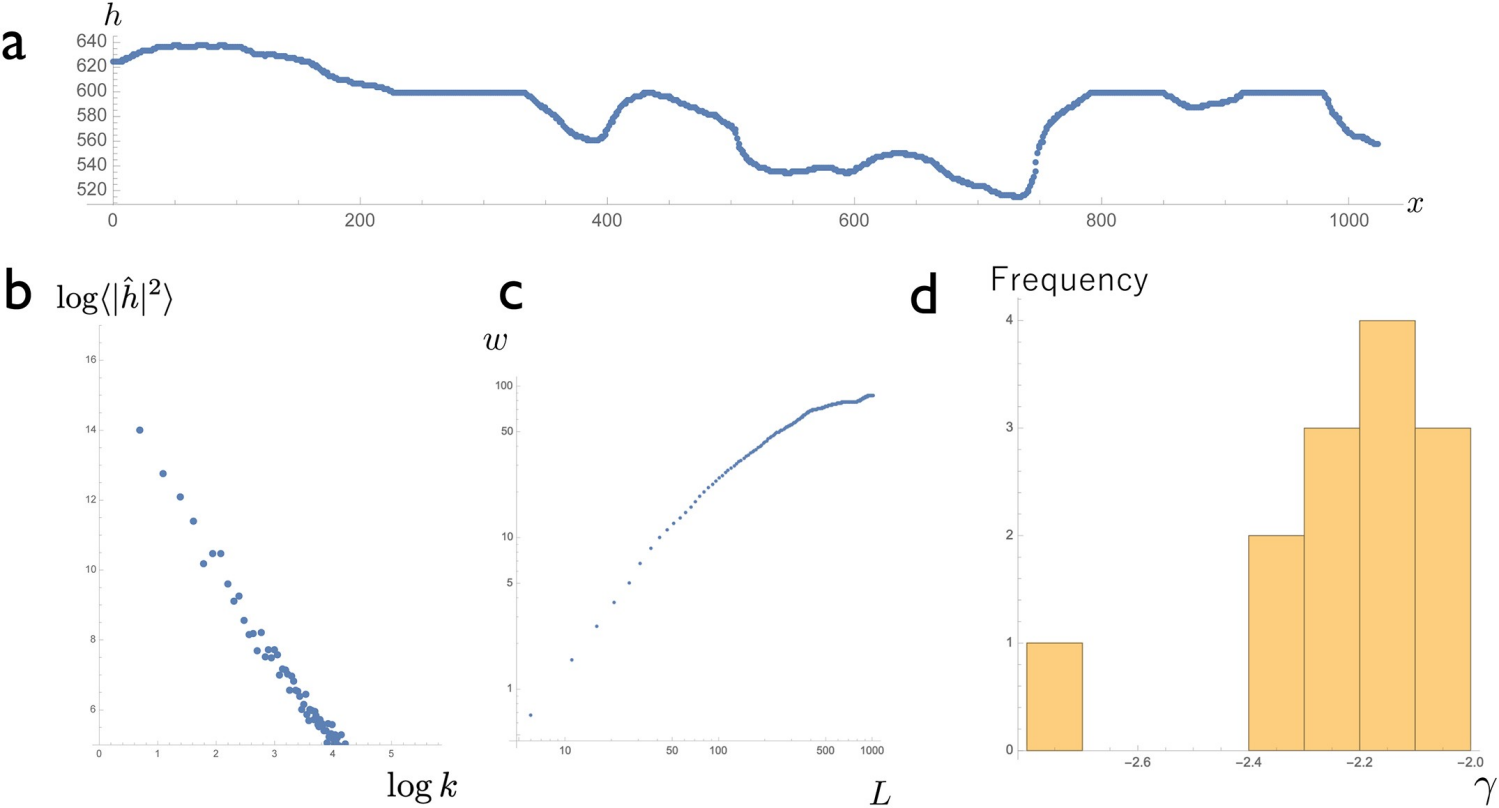
Scaling in actual skull sutures. (a) A traced suture line converted into coordinates and plotted on the *x*–*h* plane. (b) Relationship between *k* and $\langle |\hat{h}{|}^{2}\rangle$ on a log–log plot. (c) The relationship between surface roughness *w* and system size *L*. (d) Histogram of *γ* obtained by separate data.

We obtained *γ* from trace data from human sutures. Since we could not obtain a sufficient number of young skull samples for time-course analysis, we confined our analysis to the final suture forms of adult skulls. We obtained coordinate data for the anterior part of a sagittal suture (Fig 7a) and derived the power spectrum of the suture line; a log–log plots of the power spectrum showed linearity (Fig 7b), which is similar to the model behavior (Fig 5f). Scaling between surface roughness *w* and system size *L* (Fig 7c) is similar to that observed in numerical simulation (Fig 5g). The slope of the distribution was then obtained using the least-square method. This corresponded to −*γ*. The mean and standard deviation of measured *γ* were 2.2 and 0.18, respectively (Fig 7d); our model (Eq 7) was able to reproduce this scaling (Fig 5).

## Discussion

In this study, we formulated a simple model based on our previous work [19], and analytically obtained the scaling of the suture tissue, which was confirmed by experimental observations. Although many previous studies have investigated the fractality of skull sutures [8–12], ours is the first study in which the scaling observed in vivo has been directly correlated with a mathematical model of pattern formation. While previously published models have been able to generate a fractal structure, they were not fully consistent with experimental observations. For example, the Eden collision model [17, 18] did not reproduce the initial phase of pattern formation, whereas the bidirectional growth model [16] generated structures that were too complex. Moreover, neither of these previous models were analytically manageable.

In the present study, we obtained a scaling parameter *γ*, which may reflect the pattern formation mechanism. Relationship between scaling parameter and fractal dimension has been studied previously [20, 21]. Due to its high sensitivity, *γ* may be useful for diagnostic purposes. For example, in craniosynostosis, osteogenesis is promoted by constitutive active form of FGFR [2]; in the full model, this means smaller *c* value than normal. In our analysis, *γ* should become smaller by a small value of *c*. Therefore, smaller *γ* in actual suture indicates a higher risk of craniosynostosis.

In future research, the goal will be to understand scaling in larger amplitude cases. In the present study, we dealt with scaling only for sutures with a small amplitude, but there are some types of suture curvature in which the amplitude of a specific frequency is emphasized. In such cases, the system exhibits interface instability, and a positive λ_*max*_ exists, and the curvatures are much more pronounced. Our model cannot measure the scaling of sutures with such pronounced curvature and overhanging (such as lambda sutures) and can be applied only to small amplitude sutures.

Whether the fractal nature of suture tissue has a biological function remains to be elucidated. It has been postulated that the interdigitation strengthens the junction between the skull bones [5]. The functional difference between simple geometry (sine curves) and fractal structure has, however, been examined from an engineering perspective [26–28]. Although large-scale measurement of human suture strength has been done [29], the effect of interdigitation on suture strength remains unclear because the fusion of suture tissue may also influence its mechanical properties. A literature survey did not find any clear correlation between the degree of suture interdigitation and the prevalence of fractures [30]. Further study is necessary to fully understand the biological importance of the fractal nature of skull sutures.

## Supporting information

S1 Text Details of the models and mathematical analyses. (PDF)

## References

[1] Di Ieva A, Bruner E, Davidson J, Pisano P, Haider T, Stone SS, et al Cranial sutures: a multidisciplinary review. Child’s Nervous System. 2013;29(6):893–905. 10.1007/s00381-013-2061-4 23471493

[2] Cohen MM, MacLean RE. Craniosynostosis Diagnosis, Evaluation, and Management. Oxford University Press; 2000.

[3] Miura T, Perlyn CA, Kinboshi M, Ogihara N, Kobayashi-Miura M, Morriss-Kay GM, et al Mechanism of skull suture maintenance and interdigitation. Journal of Anatomy. 2009;215(6):642–655. 10.1111/j.1469-7580.2009.01148.x 19811566 PMC2796787

[4] Key CA, Aiello LC, Molleson T. Cranial suture closure and its implications for age estimation. International Journal of Osteoarchaeology. 1994;4(3):193–207. 10.1002/oa.1390040304

[5] Jaslow CR. Mechanical properties of cranial sutures. Journal of Biomechanics. 1990;23(4):313–321. 10.1016/0021-9290(90)90059-C 2335529

[6] Long CA. Intricate sutures as fractal curves. Journal of Morphology. 1985;185(3):285–295. 10.1002/jmor.1051850303 29976025

[7] Masuda Y, Yohro T. Are There Any Regularities in Cranial Sutures? Okajimas Folia Anatomica Japonica. 1987;64(1):39–45. 10.2535/ofaj1936.64.1_39 3601333

[8] Lynnerup N, Jacobsen JCB. Brief communication: Age and fractal dimensions of human sagittal and coronal sutures. American Journal of Physical Anthropology. 2003;121(4):332–336. 10.1002/ajpa.10260 12884314

[9] Yu JC, Wright RL, Williamson MA, Braselton JP, Abell ML. A Fractal Analysis of Human Cranial Sutures. The Cleft Palate-Craniofacial Journal. 2003;40(4):409–415. 10.1597/1545-1569_2003_040_0409_afaohc_2.0.co_2 12846606

[10] Monteiro LR, Lessa LG. Comparative analysis of cranial suture complexity in the genus Caiman (Crocodylia, Alligatoridae). Brazilian journal of biology = Revista brasleira de biologia. 2000;60(4):689–694. 10.1590/S0034-71082000000400021 11241970

[11] Gibert J, Palmqvist P. Fractal analysis of the Orce skull sutures. Journal of Human Evolution. 1995;28(6):561–575. 10.1006/jhev.1995.1042

[12] Long CA, Long JE. Fractal dimensions of cranial sutures and waveforms. Acta Anatomica. 1992;145(3):201–206. 10.1159/000147366 1466230

[13] Górski AZ, Skrzat J. Error estimation of the fractal dimension measurements of cranial sutures. Journal of Anatomy. 2006;208(3):353–359. 10.1111/j.1469-7580.2006.00529.x 16533317 PMC2100241

[14] Eden M. A two-dimensional growth process. Fourth Berkeley Symposium on Mathematical Statistics and Probability. 1961;4:223–239.

[15] Falconer K. Fractal Geometry. 2nd ed New York, NY: Wiley; 2003.

[16] Zollikofer CPE, Weissmann JD. A bidirectional interface growth model for cranial interosseous suture morphogenesis. Journal of Anatomy. 2011;219(2):100–114. 10.1111/j.1469-7580.2011.01386.x 21539540 PMC3162232

[17] Oota Y, Nagamine T, Ono K, Miyazima S. A two-dimensional model for sagittal suture of cranium. Forma. 2004;19(3):197–205.

[18] Oota Y, Ono K, Miyazima S. 3D modeling for sagittal suture. Physica A: Statistical Mechanics and its Applications. 2006;359(1-4):538–546. 10.1016/j.physa.2005.05.095

[19] Yoshimura K, Kobayashi R, Ohmura T, Kajimoto Y, Miura T. A new mathematical model for pattern formation by cranial sutures. Journal of Theoretical Biology. 2016;408:66–74. 10.1016/j.jtbi.2016.08.003 27519950

[20] Barabasi AL, Stanley HE. Fractal concepts in surface growth. Cambridge: Cambridge University Press; 1995.

[21] Krug J. Origins of scale invariance in growth processes. Advances in Physics. 1997;46(2):139–282. 10.1080/00018739700101498

[22] Kamioka Y, Sumiyama K, Mizuno R, Sakai Y, Hirata E, Kiyokawa E, et al Live Imaging of Protein Kinase Activities in Transgenic Mice Expressing FRET Biosensors. Cell Structure and Function. 2012;37(1):65–73. 10.1247/csf.11045 22277578

[23] Schindelin J, Arganda-Carreras I, Frise E, Kaynig V, Longair M, Pietzsch T, et al Fiji: an open-source platform for biological-image analysis. Nature Methods. 2012;9(7):676–682. 10.1038/nmeth.2019 22743772 PMC3855844

[24] Huang S. Non-genetic heterogeneity of cells in development: more than just noise. Development. 2009;136(23):3853–3862. 10.1242/dev.035139 19906852 PMC2778736

[25] Takayasu H. Fractals. Asakura Publishing Co. Ltd; 1986.

[26] Khoshhesab MM, Li Y. Mechanical behavior of 3D printed biomimetic Koch fractal contact and interlocking. Extreme Mechanics Letters. 2018;24:58–65. 10.1016/j.eml.2018.09.003

[27] Li Y, Ortiz C, Boyce MC. Bioinspired, mechanical, deterministic fractal model for hierarchical suture joints. Physical Review E—Statistical, Nonlinear, and Soft Matter Physics. 2012;85(3):1–14. 10.1103/PhysRevE.85.031901 22587117

[28] Lin E, Li Y, Weaver JC, Ortiz C, Boyce MC. Tunability and enhancement of mechanical behavior with additively manufactured bio-inspired hierarchical suture interfaces. Journal of Materials Research. 2014;29(17):1867–1875. 10.1557/jmr.2014.175

[29] Torimitsu S, Nishida Y, Takano T, Koizumi Y, Hayakawa M, Yajima D, et al Statistical analysis of biomechanical properties of the adult sagittal suture using a bending method in a Japanese forensic sample. Forensic Science International. 2015;249:101–106. 10.1016/j.forsciint.2015.01.030 25679987

[30] Ungari C, Filiaci F, Riccardi E, Rinna C, Iannetti G. Etiology and incidence of zygomatic fracture: A retrospective study related to a series of 642 patients. European Review for Medical and Pharmacological Sciences. 2012;16(11):1559–1562. 23111970

