# Supplementary material for: Noise-induced scaling in skull suture interdigitation
Source: PLoS One. 2020 Dec 17;15(12):e0235802. doi: 10.1371/journal.pone.0235802 (PMC7745973; doi:10.1371/journal.pone.0235802)
Supplement: S1 Text — (PDF) [file pone.0235802.s001.pdf]

S1 Text: Detail of the models and mathematical analyses

A. Detail of the full model (1, 2) [19].

In this section, we describe the detail of the linear dynamics of the full model (1, 2). This system has a band-like steady-state solution, but the solution can have interface instability depending on the values of parameters. Growth speed $\lambda(k)$ can be derived from linear stability analysis as follows [19]:

$$\lambda(k) = \frac{a}{\pi r^2}(\zeta(k, r) + \phi(k) - 2\sigma) - bk^2. \tag{14}$$

The functions and parameters in (14) are determined from a, b, c, and r as follows: at first we consider the steady state of the band solution without curvature (S1 Fig). Since $V = 0$ and $c = v$, we obtain

$$v = c = \frac{1}{2} - \frac{1}{\pi} \arccos \frac{2y_0}{r} + \frac{2y_0}{\pi r^2}\sqrt{r^2 - 4y_0^2} \tag{15}$$

where $2y_0$ represents the width of the band-like solution (S1 Fig). By solving (15), we obtain actual value of y_0 from c and r, which cannot be expressed in an analytically explicit form. Then, the other functions and parameters in (14) are defined using y_0 and r as follows:

$$\sigma = \sqrt{r^2 - 4y_0^2} \tag{16}$$

$$\phi(k) = \frac{2 \sin k\sigma}{k} \tag{17}$$

$$\zeta(k, r) = 2r(1 - \frac{\sin kr}{kr}). \tag{18}$$

r represents the radius of the kernel $K(x, y)$. 2σ represents the length of shorter intersection between kernel and suture interface (S1 Fig) . Phase diagram of (14) is shown in S2 Fig.

The dynamics of the interface described by equation (1) can be implemented

numerically using the phase-field method with the following Allen–Cahn equation:

$$\frac{\partial u}{\partial t} = b\Delta u + u(1-u)\left(u - \frac{1}{2} + \frac{a}{\sqrt{2b}}(c-v)\right). \tag{19}$$

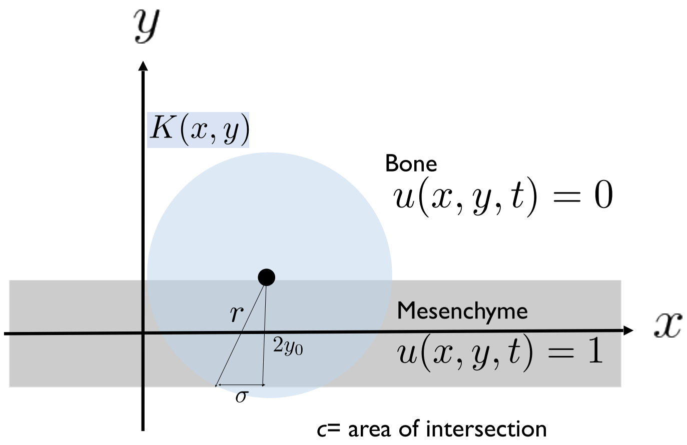

S1 **Fig. Steady state of the full model without curvature.**

B. Explicit form of $\mathscr{L}$

From the equation (14), the Fourier transformation of $\mathscr{L}(h(x,t))$ can be represented as

$$\widehat{\mathscr{L}(h)}(k,t) = \lambda(k)\hat{h}(k,t) = \left\{-bk^2 + \frac{2a(r-\sigma)}{\pi r^2} + \frac{a}{\pi r^2}\left(\frac{2\sin k\sigma}{k} - \frac{2\sin kr}{k}\right)\right\}\hat{h}(k,t).$$

Here, we note that the Fourier transform is defined as

$$\hat{h}(k,t) = \int_{-\infty}^{\infty} h(x,t)e^{-ixk}dy$$

in this paper. Then, we can obtain that

$$\widehat{(\Delta h)}(k,t) = -k^2\hat{h}(k,t), \quad \widehat{(L*h)}(k,t) = \hat{L}(k)\hat{h}(k,t) = \left(\frac{2\sin k\sigma}{k} - \frac{2\sin kr}{k}\right)\hat{h}(k,t),$$

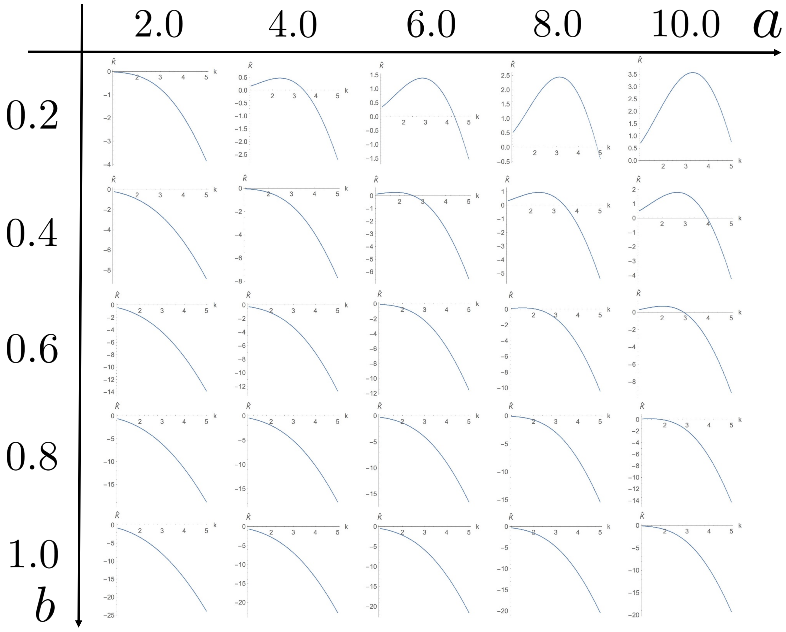

S2 **Fig. Phase diagrams for the dispersion relation of the full model with systematic changes in a and b.** In the present study, we concentrated on the parameter space in which the growth speed $\lambda(k) < 0$ for all k (lower left parameter sets).

where $*$ represents convolution defined as

$$(L * h)(x, t) = \int_{-\infty}^{\infty} L(x - y)h(y, t)dx$$

and $L(x)$ is a convolution kernel defined by the following step function:

$$L(x) = -\frac{a}{\pi r^2}\chi_{\sigma,r}(x), \quad \chi_{\sigma,r}(x) = \begin{cases} 1 & \sigma < |x| < r \\ 0 & \text{otherwise} \end{cases}.$$

Thus, the linear operator $\mathscr{L}$ can be described explicitly by inverse Fourier transformation of $\lambda(k)$ as follows:

$$\mathscr{L}(h(x, t)) = b\Delta h(x, t) + \frac{2a(r - \sigma)}{\pi r^2}h(x, t) + (L * h)(x, t).$$

C. Scaling: Power spectrum $\langle|\hat{h}|^2\rangle \propto k^{-\gamma}$

Fractal patterns should obey the rule $|\hat{h}|^2 \propto k^{-\gamma}$ in the frequency domain [25], where $|\hat{h}|^2$ is the power spectrum of the interface shape and k is the wavenumber. An intuitive explanation of this is as follows. The fractal structure should remain self-similar even when the spatial scale for observation is changed. The observation scale can be regarded as a cutoff value in the frequency domain. The function should be in the form $|\hat{h}|^2 \propto k^{-\gamma}$ to retain the similarity of the power spectrum when the cutoff value is changed (S3 Fig in S1 text).

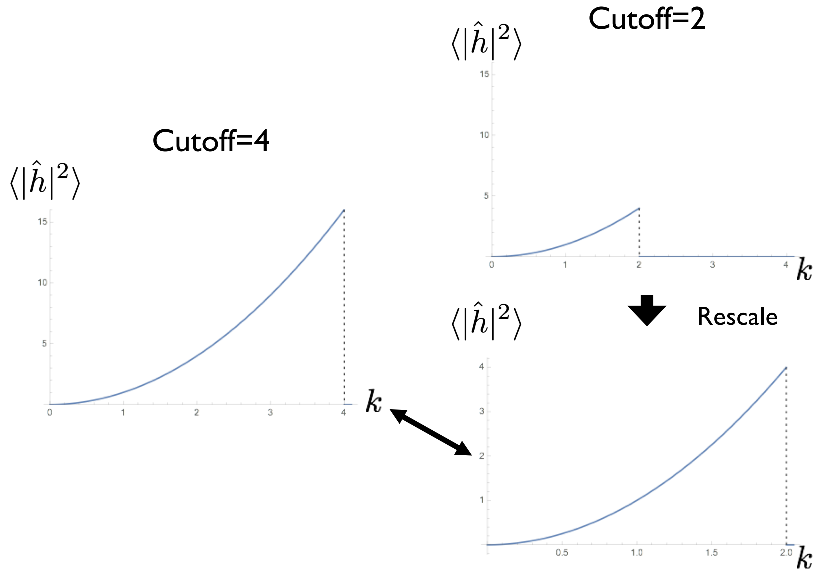

S3 **Fig. Relationship between the wavenumber and amplitude of fractal structures.** A fractal structure should exhibit self-similarity in the frequency domain. Measurement at a certain spatial scale means cutting off the structure smaller than a certain threshold. Therefore, in a fractal structure, the power spectrum should be similar across all cutoff values. This is satisfied when $\hat{h} \propto k^{-\gamma}$.

D. Movement of the band-like solution is proportional to the *gradient* of v perturbation

In this section, we consider how the suture tissue moves according to the external perturbation of v in the full model. In this study, we introduced a noise term, which

should affect the movement of two wavefronts. At first we consider the straight suture tissue in the full model (1, 2) (S4 Fig). In this setting, the speeds of the wavefronts V_1 and V_2 are defined as follows:

$$V_1 = a(c - v(x, y_0)) = -av(x, y_0) + ac \tag{20}$$

$$V_2 = a(c - v(x, -y_0)) = -av(x, -y_0) + ac \tag{21}$$

$$v = K * u + \bar{g}y \tag{22}$$

where a is the efficiency of the substrate factor (the osteogenesis-promoting diffusible signaling molecules expressed at the mesenchyme) over bone differentiation, and c is the threshold value for bone generation/resorption. Since we consider straight line, we set surface tension $b = 0$. $v(x, y)$ represents the effect of the substrate factor, determined by the convolution of the kernel $K(x, y)$ and bone shape $u(x, y)$ (with $u(x, y) = 0$ representing the bone and $u(x, y) = 1$ representing the mesenchyme).

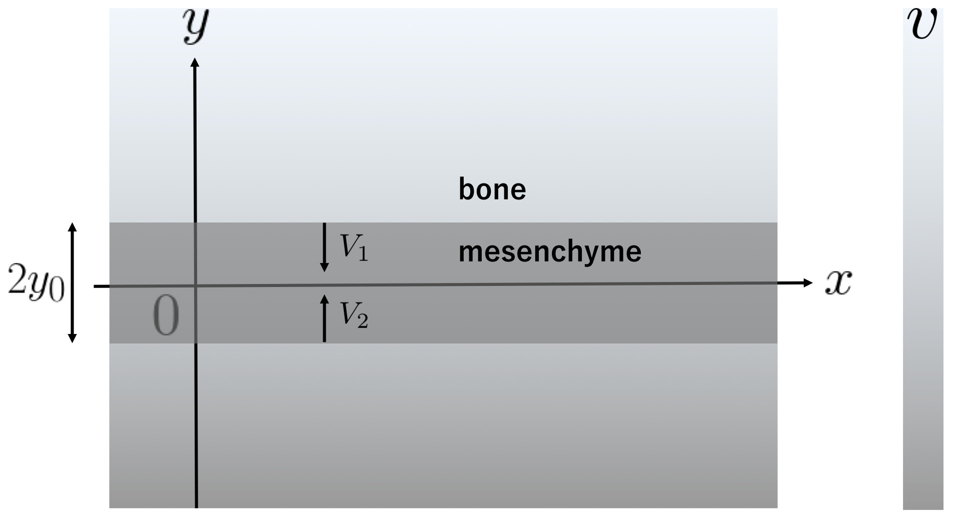

S4 **Fig. Parameter definitions for the mathematical analysis of the band-like solution movement.** The original model [19] considered band-like solutions with width $2y_0$. The present model focuses on the dynamics of the centerline of this band-like solution $h(x)$. We only considered the onset of pattern formation without overhangs.

We initially considered the steady state without a gradient resulting from a noise term ($\bar{g} = 0$). And since we consider steady state, $V_1 = V_2 = 0$. Then, from (20, 21) we obtain

$$0 = -aK * u(x, y_0) + ac \tag{23}$$

$$0 = -aK * u(x, -y_0) + ac. \tag{24}$$

Next, we consider the effect of noise (5, 6). $H(x, y, t)$ is the time–space-dependent noise of v, and we define $\bar{g}$ as the noise-induced gradient of v in the y direction:

$$\bar{g} = \frac{\partial H(x, y, t)}{\partial y}. \tag{25}$$

. Then we considered the movement of V_1 and V_2 with $\bar{g}$ resulting from H.

$$V_1 = -av(x, y_0) + ac = -a\bar{g}y_0 \tag{26}$$

$$V_2 = -av(x, -y_0) + ac = a\bar{g}y_0. \tag{27}$$

Thus, the band moves at speed $a\bar{g}y_0$ without changing its width. We confirmed by numerical simulation of the model (5, 6) with various external y-directional gradient that the band solution moved according to the gradient of v (S5 Fig).

In the reduced model, the movement of $h(x, t)$ is described as follows:

$$\frac{\partial h(x, t)}{\partial t} = \mathscr{L}(h(x, t)) + ay_0 \frac{\partial H(x, y, t)}{\partial y}. \tag{28}$$

Thus, the velocity of suture line movement is proportional to the gradient of H in the y-direction. We also confirmed numerically that η can be regarded as a white noise (S6 Fig.

E. Scaling of the dispersion relation at very large and small spatial scales

For very large and small spatial scales at which the effect of the kernel is negligible, the dispersion relation relies purely on surface tension. At an extremely large or small

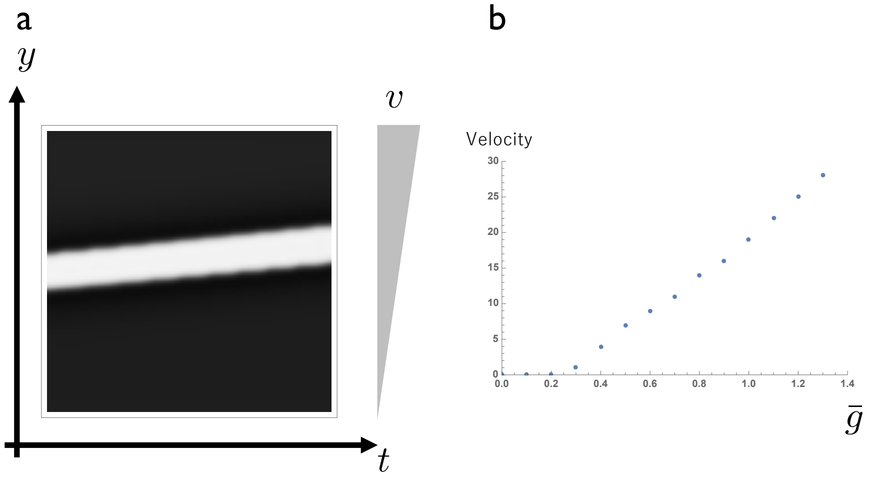

S5 **Fig. Movement of the band-like solution was dependent on the gradient of v.** (a) Simulation result of the band-like solution movement according to the external gradient of v. The solution moves at a constant speed. (b) Relationship between the external gradient $\bar{g}$ and the velocity of the band solution movement. The velocity was proportional to $\bar{g}$.

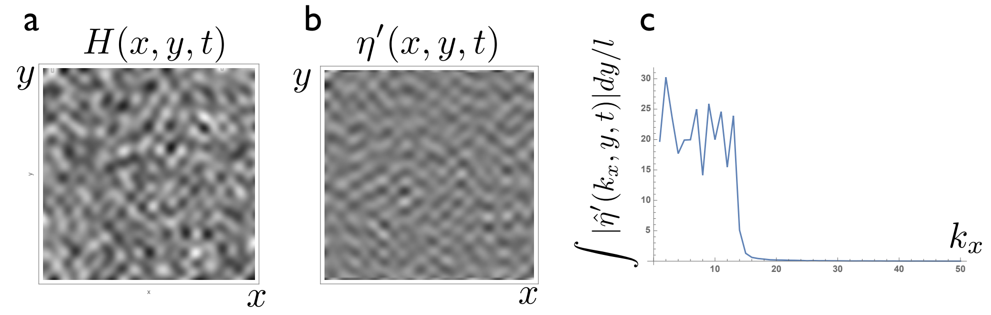

S6 **Fig. Relationship between** $H(x, y, t)$ **and** $\eta(x, t)$. (a) The distribution of noise ($H(x, y, t)$) in the full model. (b) The noise term of the reduced model. At first we defined $\eta'(x, y, t) = \partial H/\partial y$, and defined noise term in the reduced model as $\eta(x, t) = \eta'(x, h(x), t)$. (c) The frequency-domain $\hat{\eta}(k_x, y, t)$ in the x-direction. This can be considered to be white noise in the x-direction.

spatial scale, $\lambda(k) \propto -k^2$, and $\lambda(k)$ can be expressed as

$$\lambda(k) = -bk^2 + \frac{2a}{\pi r^2}\left(r - \sigma - (\frac{\sin rk}{k} - \frac{\sin \sigma k}{k})\right). \tag{29}$$

When k is large, $-bk^2$ becomes dominant. When k is small,

$$\lambda(k) = -bk^2 + \frac{2a}{\pi r^2}\left(r - \sigma - (\frac{\sin rk}{k} - \frac{\sin \sigma k}{k})\right) \approx \left(-b + \frac{a}{3\pi r^2}(r^3 - \sigma^3)\right)k^2 + O(k)^4, \tag{30}$$

thus $\lambda(k) \propto k^2$. If we observe a wide range of scaling in $\lambda(k)$, there are three regions of different scaling. The scaling we are interested in is in the region around $k = 1$. In the other two regions, the scaling is always k^2 and independent of the model parameters (S7 Fig in S1 text).

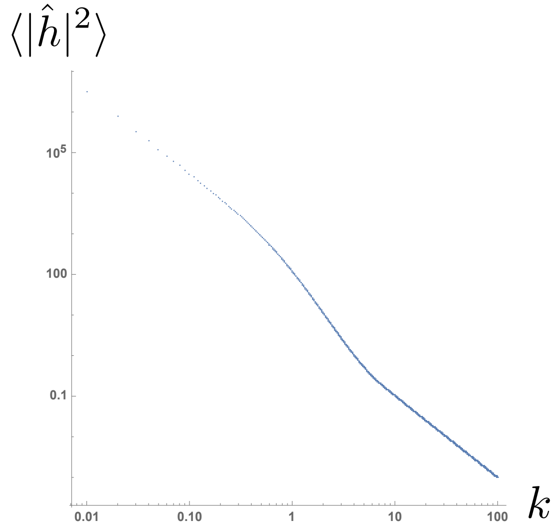

S7 **Fig. Scaling of** $\langle\,\hat{h}\ (k,\infty)^2\rangle$ **across a wide range.** Three independent linear regions are observed. In regions $k < 0.1$ and $k > 10$ the scaling is independent of the model parameters. We are interested in the region around $k = 1$. The model parameters are $r = 1$, $a = 1$, $b = 0.1$, and $c = 0.48$.
